# Solvent Polarity Shapes Antioxidant Capacity, Preliminary Hyaluronidase Inhibition, and Chemical Profile of *Buddleja officinalis* Extracts

**DOI:** 10.3390/molecules31101706

**Published:** 2026-05-18

**Authors:** Gang Tian, Yihang Tian, Shiping Cheng, Cong Yang, Yongjun Han

**Affiliations:** 1 School of Chemistry and Chemical Engineering, Pingdingshan University, Pingdingshan 467000, China; 18639020759@163.com (Y.T.); shipingcheng@163.com (S.C.); hyj73@126.com (Y.H.); 2 Henan Key Laboratory of Germplasm Innovation and Utilization of Eco-Economic Woody Plant, Pingdingshan 467000, China; 3 Yaoshan Laboratory, Pingdingshan University, Pingdingshan 467000, China; 4 School of Mathematics and Statistics, Pingdingshan University, Pingdingshan 467000, China; yangcong996@126.com

**Keywords:** *Buddleja officinalis*, solvent polarity, chemical antioxidant capacity, hyaluronidase inhibition, flavonoid glycosides, UPLC–QTOF–MS

## Abstract

This study comparatively evaluated how solvent polarity affects the chemical antioxidant capacity, preliminary hyaluronidase inhibition, and supportive chemical features of *Buddleja officinalis* Maxim. Six extracts prepared with petroleum ether, ethyl acetate, *n*-butanol, water, 60% ethanol, and 95% ethanol were assessed using DPPH·, ABTS^+^·, ferric reducing power, and hyaluronidase inhibition assays, together with total flavonoid and linarin determinations. The 60% ethanol extract showed the strongest overall radical-scavenging activity, reaching 95% DPPH· scavenging and 95% ABTS^+^· scavenging at 2 mg/mL, whereas the 95% ethanol extract showed the highest ferric reducing power under the tested conditions. Total flavonoid and linarin contents were highest in the ethanol-rich fractions, especially the 95% ethanol and 60% ethanol extracts. In the hyaluronidase assay, both the petroleum ether and 60% ethanol extracts showed relatively strong inhibition at 2.5 mg/mL, with inhibition rates of 74% and 68%, respectively, suggesting that different chemical classes may contribute to this endpoint. Supportive ^1^H NMR and UPLC–QTOF–MS data indicated clear polarity-dependent compositional differences; the 60% ethanol extract was enriched in phenylethanoid glycosides and flavonoid glycosides, whereas the petroleum ether extract showed predominantly lipophilic features. Overall, medium-polarity phenolic-rich fractions were more closely associated with chemical antioxidant capacity, while hyaluronidase inhibition may involve contributions from both non-polar and medium-polarity constituents. The present extract-level comparison provides a useful basis for fraction selection, extract standardization, and follow-up activity-guided studies.

## 1. Introduction

*Buddleja officinalis* Maxim. is a medicinal and edible plant of interest for comparative extract-level study because solvent polarity may strongly influence extract composition and observed bioactivities. Recent reviews indicate that *B. officinalis* contains flavonoids, phenylethanoid glycosides, and other secondary metabolites associated with diverse pharmacological activities [1]. Among these reported bioactivities, antioxidant-related effects and chemical antioxidant capacity have most consistently been associated with flavonoid-rich or ethanol-derived fractions, but comparatively less attention has been given to how solvent polarity shapes extract-level differences in chemical antioxidant capacity, hyaluronidase inhibition, and supportive chemical features within a unified experimental framework.

Solvent polarity is a major determinant of extract composition because phenolic acids, flavonoid glycosides, phenylethanoid glycosides, terpenoids, lipids, and other secondary metabolites differ markedly in solubility and partitioning behavior. Consequently, extracts prepared with solvents of different polarity may show distinct chemical antioxidant capacity, enzyme inhibition, and other in vitro functional properties even when derived from the same plant material. For medicinal plants with chemically diverse constituents, comparative extract-level studies are therefore useful for identifying bioactivity-associated fractions before compound-level isolation and mechanism-oriented evaluation.

Oxidative stress is closely involved in the development of diabetes, vascular injury, and other chronic disorders [2,3,4,5]. Earlier studies have shown that extraction conditions and isolated constituents of *B. officinalis* are associated with antioxidant-related and anti-inflammatory effects [6,7]. In the present study, DPPH·, ABTS^+^·, and ferric reducing power assays were used to compare assay-based radical-scavenging and reducing responses among extracts; therefore, the results are described as chemical antioxidant capacity under defined in vitro conditions [8].

In addition to antioxidant effects, *B. officinalis* has also been linked to anti-inflammatory activity in dry-eye-related models, including effects on lacrimal gland apoptosis and inflammatory factors, and aqueous extracts have shown activity against vascular inflammatory responses in human umbilical vein endothelial cells [9,10,11,12]. Hyaluronidase is a biologically relevant target in this context because it degrades hyaluronic acid and contributes to inflammatory and allergic processes [13,14]. Plant-derived hyaluronidase inhibitors have also attracted attention as potential anti-inflammatory, anti-allergic, and skin-protective agents, although inhibitory results from crude extracts require careful interpretation because assay conditions, reference inhibitors, and IC_50_ determinations strongly influence comparability [15,16]. Even so, hyaluronidase inhibition by polarity-differentiated extracts of *B. officinalis* has not been systematically compared.

Against this background, the present work was designed as a comparative extract-level study to clarify how solvent polarity influences chemical antioxidant capacity, preliminary hyaluronidase inhibition, and supportive chemical features in *B. officinalis*. Six extracts prepared with petroleum ether, ethyl acetate, *n*-butanol, water, 60% ethanol, and 95% ethanol were compared within a unified experimental framework. Chemical antioxidant capacity was evaluated by DPPH· radical-scavenging, ABTS^+^· radical-scavenging, and ferric reducing power assays, whereas hyaluronidase inhibition was assessed using a modified Elson–Morgan method. In parallel, total flavonoid and linarin contents were determined, and supportive compositional evidence was obtained from ^1^H NMR and UPLC–QTOF–MS profiling. The aim of the study was not to assign activity definitively to individual molecules, but rather to establish an extract-level evidence chain linking bioactivity differences, composition–activity associations, and representative chemical characteristics, thereby providing a more informative basis for fraction selection and subsequent follow-up studies.

## 2. Materials and Methods

### 2.1. Materials and Instruments

Dried flower buds of *B. officinalis* were obtained from Qingchuan, Guangyuan, Sichuan, China, powdered, and passed through a 60-mesh sieve before extraction. The plant material was authenticated by Prof. Shiping Cheng, and a voucher specimen (No. BO-2025-0617) was deposited at Yaoshan Laboratory, Pingdingshan University. DPPH· (Sigma-Aldrich, St. Louis, MO, USA); Methanol (HPLC grade), ethyl acetate, and *n*-butanol (Beijing Bailingwei Technology Co., Ltd., Beijing, China); L-ascorbic acid, ABTS, and other analytical-grade reagents were purchased from Sinopharm Chemical Reagent Co., Ltd., Shanghai, China; Potassium persulfate (Beijing Ouhe Technology Co., Ltd., Beijing, China); Phosphate-buffered saline (PBS) (Meilun Biotechnology Co., Ltd., Dalian, China); Potassium ferricyanide (Solarbio Science & Technology Co., Ltd., Beijing, China); Trichloroacetic acid and absolute ethanol (Aladdin Biochemical Technology Co., Ltd., Shanghai, China); Ferric chloride (Beijing Chemical Works, Beijing, China). Rutin reference standard was obtained from the National Institutes for Food and Drug Control (Beijing, China), and linarin reference standard (>98%) was obtained from Macklin Biochemical Co., Ltd. (Shanghai, China). Main instruments included a Microplate Reader (BioTek Instruments, Inc., Winooski, VT, USA, Model SLXFATS), a Waters e2695 HPLC system (Waters Corporation, Milford, MA, USA), a Bruker AVANCE 400 MHz NMR spectrometer (Bruker BioSpin GmbH, Rheinstetten, Germany), and a Waters ACQUITY H-Class UPLC coupled to a Xevo G2-XS QTOF mass spectrometer (Waters Corporation, Milford, MA, USA).

### 2.2. Preparation of Solvent Extracts

Powdered *B. officinalis* material (100 g each) was extracted separately with petroleum ether, ethyl acetate, *n*-butanol, 95% ethanol, 60% ethanol, or water. For each solvent, the extraction was performed independently in triplicate. For each extraction, the solid-to-liquid ratio was 1:7 (*w*/*v*; 100 g plant material with 700 mL solvent). Samples were sonicated at 50 °C for 1 h and then macerated at room temperature for 12 h without mechanical agitation. After filtration, the filtrates were concentrated under reduced pressure to obtain crude extracts and then freeze-dried. The dried extracts were stored until analysis. All reported values represent the mean ± SD of three independently prepared extracts. All bioactivity and compositional results were compared on an equal dry-extract mass basis. Therefore, the results reflect the functional and chemical characteristics of the dried extracts themselves rather than the total recovery of active constituents from the original plant material.

### 2.3. Determination of Total Flavonoid Content

Total flavonoids were selected as representative compositional indicators because flavonoids are among the major reported constituents of *B. officinalis* and are closely related to its phenolic-rich chemical profile. Linarin was further quantified as a representative flavonoid glycoside marker of *B. officinalis*. Total phenolic content was not determined in the present design; therefore, the composition–activity analysis should be interpreted as being based on selected compositional indicators rather than a complete phenolic characterization.

Total flavonoid content was determined using a rutin-based colorimetric method. A rutin calibration curve was prepared over the linear range of 0.039–2.5 mg/mL, yielding the regression equation A = 1.2753C + 0.0527 with R^2^ = 0.9998. For sample analysis, 20 mg of dried extract was dissolved in methanol and diluted to 10 mL. The solution was sonicated and centrifuged, and the clear supernatant was used for analysis. Although the same redissolution procedure was applied to all extracts, differences in redissolution efficiency among extracts of different polarity cannot be completely excluded and may influence the apparent comparability of the results on a dry-extract basis. After sonication and centrifugation, aliquots were reacted sequentially with NaNO_2_, Al(NO_3_)_3_, and NaOH, and absorbance was measured at 510 nm. Results were expressed as mg rutin equivalents per g dry extract. The colorimetric determination of total flavonoids was performed with reference to previously reported rutin-based spectrophotometric procedures [17].

### 2.4. Quantification of Linarin

Linarin was quantified by HPLC using a Waters Symmetry C18 column (4.6 × 250 mm, 5 μm) at 40 °C. The mobile phase consisted of methanol–water–acetic acid (45:54.5:0.5, *v*/*v*/*v*), delivered at 0.8 mL/min. The detection wavelength was 326 nm and the injection volume was 10 μL. The linarin calibration curve was Y = 2.0 × 10^7^X − 6794.8 with R^2^ = 0.9999 over the concentration range of 5–50 μg/mL. Linarin content was reported as mg/g dry extract.

### 2.5. DPPH· Radical-Scavenging Assay

For the DPPH· assay, all dried extracts were re-dissolved in methanol, sonicated to assist dissolution, centrifuged when necessary, and diluted to the required concentrations using the same solvent system. The corresponding solvent blank was included to correct for background absorbance and possible solvent effects.

A 1 mmol/L DPPH· solution was prepared in methanol. Each extract was dissolved at 2 mg/mL and serially diluted to 0.125 mg/mL. For analysis, 200 μL of sample solution was mixed with 600 μL of DPPH· solution and allowed to react in the dark for 30 min at room temperature. Absorbance was measured at 517 nm. VC was tested under the same concentration series as the positive control. Scavenging percentage was calculated using sample, solvent, and reagent blank corrections.

### 2.6. ABTS^+^· Radical-Scavenging Assay

ABTS^+^· working solution was prepared by reacting ABTS with potassium persulfate and then diluting the resulting stock solution to the required working concentration. VC was used as the positive control under the same assay conditions. Extracts and VC were tested over the same concentration range used in the DPPH· assay. The extract stock solutions used for the ABTS^+^· assay were prepared using the same re-dissolution procedure as described for the DPPH· assay, and solvent blanks were included for correction. In each test, 200 μL of ABTS^+^· working solution was mixed with 10 μL of sample solution, incubated in the dark for 30 min, and the absorbance was measured at 405 nm using a microplate reader. In the present manuscript, ABTS^+^· results are presented as relative scavenging percentages for consistency with the concentration-dependent comparison across solvent fractions.

### 2.7. Ferric Reducing Power Assay

Ferric reducing power was determined using potassium ferricyanide reduction followed by ferric chloride color development. One millilitre of sample solution was mixed with 2.5 mL of 0.2 mol/L phosphate buffer and 2.5 mL of 1% potassium ferricyanide, incubated at 50 °C for 20 min, treated with 10% trichloroacetic acid, and centrifuged. The supernatant was then mixed with ethanol and 0.1% ferric chloride, and absorbance was measured at 700 nm. The blank-corrected absorbance was used as the reducing power index.

### 2.8. Hyaluronidase Inhibitory Activity

Hyaluronidase inhibition was measured using a modified Elson–Morgan method. Extract solutions at 2.5, 5.0, and 7.5 mg/mL were incubated with hyaluronidase in acetate buffer, followed by CaCl_2_, sodium hyaluronate, NaOH, acetylacetone reagent, Ehrlich’s reagent, and ethanol according to the established procedure. Absorbance was measured at 530 nm, and inhibition percentage was calculated using systems with and without enzyme and sample. Because this assay was designed as a preliminary comparative screening among solvent fractions rather than a benchmarked potency assay, no positive inhibitor control or IC_50_ determination was included in the present experimental design. Therefore, the results are reported only as preliminary comparative inhibition percentages and should not be interpreted as definitive inhibitory potency.

### 2.9. ^1^H NMR Analysis

^1^H NMR spectra were recorded on a Bruker AVANCE 400 MHz NMR spectrometer (Bruker BioSpin GmbH, Rheinstetten, Germany) at 297.3 K using DMSO-*d*_6_ as the solvent. Spectra were acquired for aqueous, 60% ethanol, 95% ethanol, ethyl acetate, *n*-butanol, and petroleum ether extracts. Experimental parameters were as follows: pulse sequence, zg30; number of scans, 8; relaxation delay, 1.0 s; acquisition time, 2.04 s. The NMR data were used only as supportive class-level compositional evidence to compare broad chemical features among extracts of different polarity, rather than for full metabolite annotation, semi-quantitative comparison, or direct activity attribution.

### 2.10. UPLC–QTOF–MS Profiling and Tentative Compound Identification

Chemical profiling was carried out on a Waters ACQUITY H-Class UPLC system coupled to a Waters Xevo G2-XS QTOF mass spectrometer (Waters Corporation, Milford, MA, USA) with an electrospray ionization source. Separation was achieved on a Waters BEH C18 column (2.1 × 100 mm, 1.7 μm) at 40 °C. The mobile phase consisted of 0.1% formic acid in water (A) and 0.1% formic acid in acetonitrile (B) at a flow rate of 0.4 mL/min. The gradient program was 0–1 min, 5% B; 1–35 min, 5–98% B; 35–37 min, 98% B; and 37.1–40 min, 5% B. The injection volume was 2 μL. Data were acquired in both ESI^+^ and ESI^−^ modes over *m*/*z* 50–1200. Compounds were tentatively assigned using accurate mass, isotopic pattern, MS/MS fragmentation, and comparison with the Waters Traditional Medicine Library 2.0 and relevant literature [18]. Only linarin and verbascoside (acteoside) were supported by comparison with authentic reference standards; all other identifications remain tentative. It should be noted that ESI-based UPLC–QTOF–MS preferentially detects polar or readily ionizable constituents. Therefore, non-polar or weakly ionizable compounds, especially those potentially enriched in the petroleum ether extract, may be underrepresented under the current MS conditions.

### 2.11. Data Processing and Correlation Analysis

Unless otherwise stated, all quantitative results are expressed as mean ± SD from three independently prepared extracts per solvent (*n* = 3). Origin 2019b was used for figure preparation, and SPSS 20.0 was used for Pearson correlation analysis. Because the correlation analysis was based on only six solvent fractions, these relationships were treated as exploratory associations rather than inferential evidence of causal contribution.

## 3. Results

### 3.1. Total Flavonoid Content of Extracts Prepared with Solvents of Different Polarity

**Figure 1:**
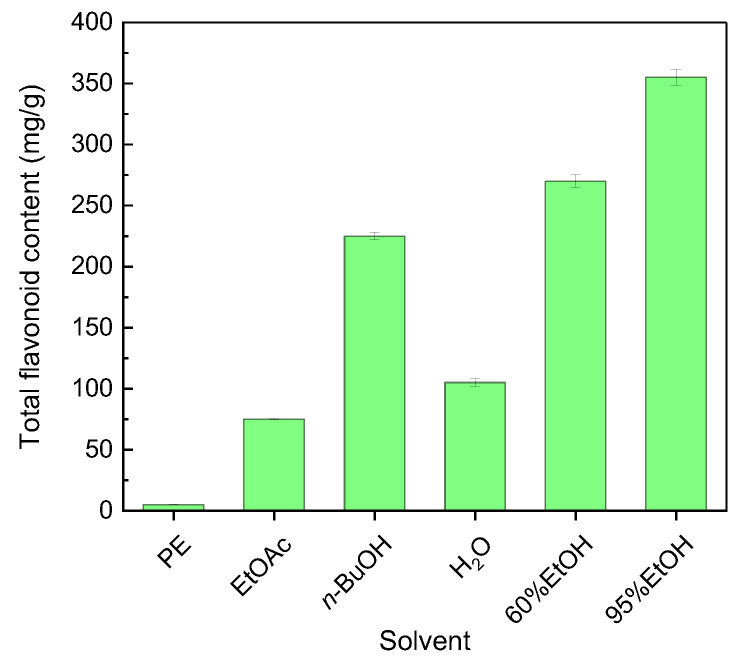
Total flavonoid content in different solvent extracts of *B. officinalis*. PE, petroleum ether; EtOAc, ethyl acetate; *n*-BuOH, *n*-butanol; H_2_O, water; 60%EtOH, 60% ethanol; 95%EtOH, 95% ethanol. Values are mean ± SD (*n* = 3).

The total flavonoid contents of the six solvent extracts of *B. officinalis* are shown in Figure 1. The 95% ethanol, 60% ethanol, and n-butanol extracts showed the highest total flavonoid contents, reaching 355 ± 6.223, 270 ± 5.176, and 225 ± 3.007 mg/g dry extract, respectively. The water extract contained 105 ± 3.1098 mg/g, whereas the ethyl acetate and petroleum ether extracts contained much lower levels, at 75 ± 0.3224 and 5 ± 0.0216 mg/g, respectively. The overall order was 95%EtOH > 60%EtOH > *n*-BuOH > H_2_O > EtOAc > PE. These data indicate that medium- and high-polarity solvents extracted flavonoid-rich fractions more efficiently on a dry-extract basis. However, because the comparison was made on a dry-extract mass basis, these results should not be interpreted as indicating the total flavonoid recovery from the original plant material.

### 3.2. Linarin Content of Extracts Prepared with Solvents of Different Polarity

**Figure 2:**
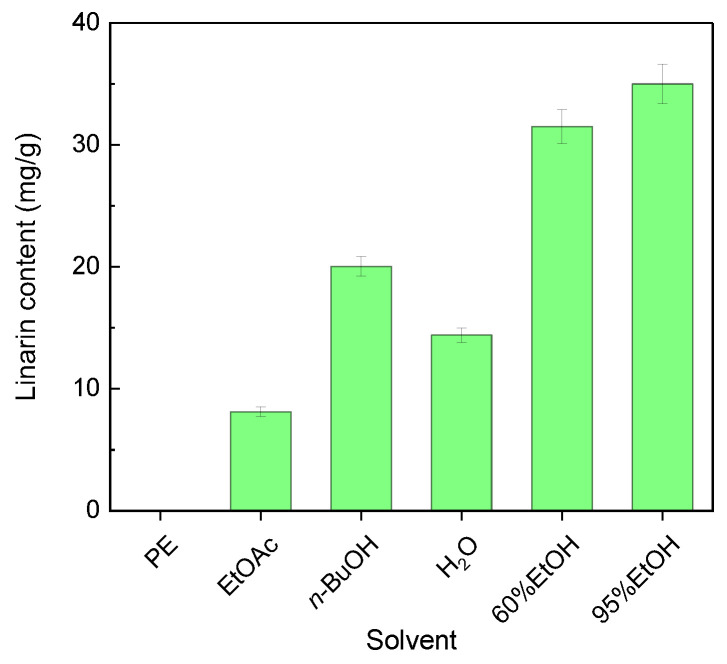
Linarin content in different solvent extracts of *B. officinalis*. Abbreviations are the same as in Figure 1. Values are mean ± SD (*n* = 3).

The linarin contents of the six solvent extracts are summarized in Figure 2. The 95% ethanol extract had the highest linarin content (34.997 ± 1.6275 mg/g dry extract), followed by the 60% ethanol extract (31.5 ± 1.37 mg/g) and the *n*-butanol extract (20.03 ± 0.7932 mg/g). The water and ethyl acetate extracts contained intermediate levels, at 14.4 ± 0.59328 and 8.11 ± 0.3876 mg/g, respectively, whereas the petroleum ether extract contained only a trace amount (0.51 ± 0.0255 mg/g). The rank order was therefore 95%EtOH > 60%EtOH > *n*-BuOH > H_2_O > EtOAc > PE, which was broadly consistent with the distribution pattern of total flavonoids.

### 3.3. Chemical Antioxidant Capacity of the Solvent Extracts

The chemical antioxidant capacities of the solvent extracts were evaluated using DPPH· radical-scavenging, ABTS^+^· radical-scavenging, and ferric reducing power assays. Overall, the 60% ethanol and 95% ethanol extracts showed stronger antioxidant capacity than the other fractions, whereas the petroleum ether extract showed consistently weak activity in the radical-scavenging capacity.

#### 3.3.1. DPPH· Radical-Scavenging Activity

**Figure 3:**
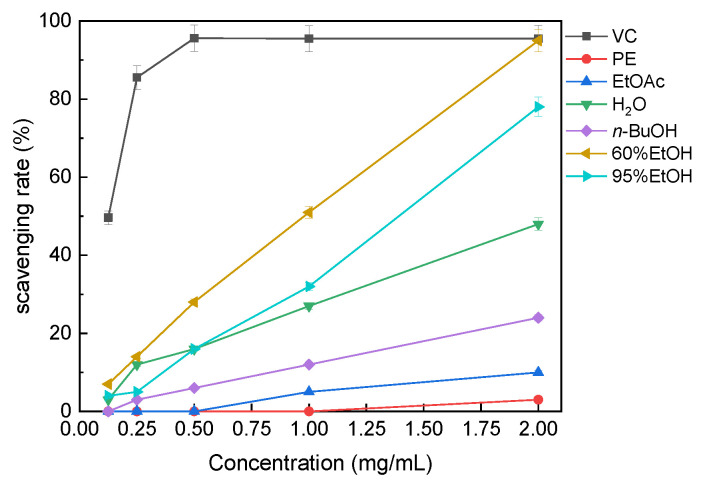
DPPH· radical-scavenging activity of solvent extracts of *B. officinalis*. VC, vitamin C; PE, petroleum ether; EtOAc, ethyl acetate; *n*-BuOH, n-butanol; H_2_O, water; 60%EtOH, 60% ethanol; 95%EtOH, 95% ethanol. Values are mean ± SD (*n* = 3).

The DPPH· radical-scavenging capacities of the solvent extracts are shown in Figure 3. The DPPH· scavenging response of all extracts increased with increasing concentration. Among the tested fractions, the 60% ethanol extract showed the strongest response, reaching approximately 95% scavenging at 2 mg/mL. The 95% ethanol extract also showed strong scavenging capacity, followed by the water and *n*-butanol extracts. In contrast, the ethyl acetate and petroleum ether extracts showed lower DPPH· scavenging capacity under the tested conditions. Based on the tested concentration range, the overall order of DPPH· radical-scavenging capacity was 60%EtOH > 95%EtOH > H_2_O > *n*-BuOH > EtOAc > PE.

#### 3.3.2. ABTS^+^· Radical-Scavenging Activity

**Figure 4:**
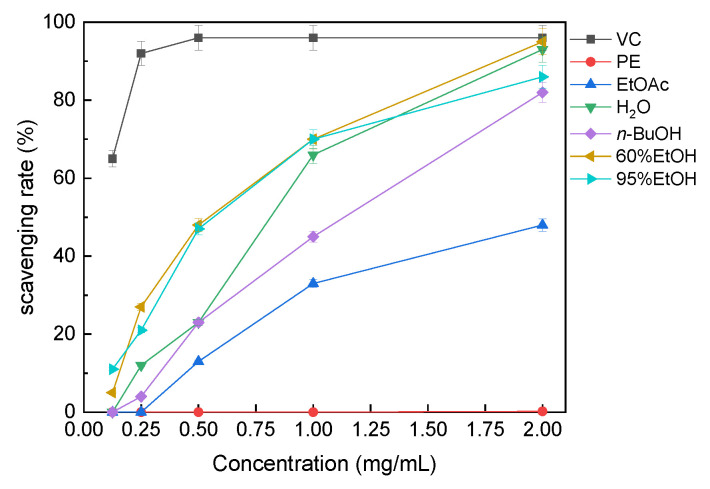
ABTS^+^· radical-scavenging activity of solvent extracts of *B. officinalis*. Abbreviations are the same as in Figure 3. Values are mean ± SD (*n* = 3).

The ABTS^+^· radical-scavenging capacities of the solvent extracts are shown in Figure 4. The ABTS^+^· scavenging response also increased with increasing concentration. At 2 mg/mL, the 60% ethanol extract reached approximately 95% scavenging, showing the strongest response among the solvent fractions. The 95% ethanol extract also showed strong ABTS^+^· scavenging capacity, whereas the petroleum ether extract showed the weakest response. These results were broadly consistent with the DPPH· assay and suggest that hydroalcoholic extracts, especially the 60% ethanol fraction, have stronger radical-scavenging capacity under the tested conditions.

#### 3.3.3. Ferric Reducing Power

**Figure 5:**
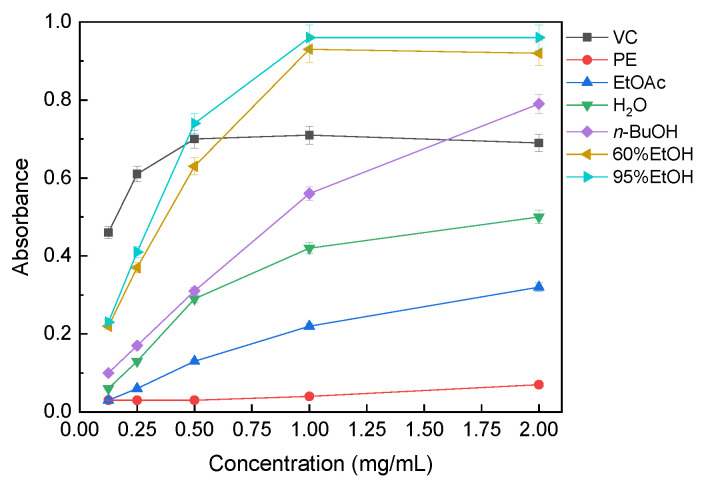
Ferric reducing power of solvent extracts of *B. officinalis*. Abbreviations are the same as in Figure 3. Values are mean ± SD (*n* = 3).

The ferric reducing powers of the solvent extracts are presented in Figure 5. The ferric reducing power assay also showed concentration-dependent behavior. At 2 mg/mL, the 95% ethanol extract showed the highest absorbance (0.96), followed closely by the 60% ethanol extract (0.92). The *n*-butanol extract also exhibited relatively strong reducing power (0.79), whereas the water extract showed moderate activity (0.50). The ethyl acetate and petroleum ether extracts displayed much lower reducing ability, with absorbance values of 0.32 and 0.07, respectively. Therefore, under the present assay conditions, the reducing power ranking was 95%EtOH > 60%EtOH > *n*-BuOH > H_2_O > EtOAc > PE.

### 3.4. Hyaluronidase Inhibitory Activity of the Solvent Extracts

**Figure 6:**
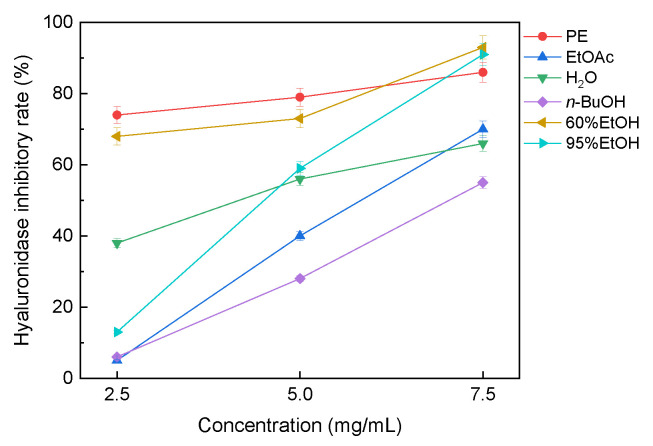
Hyaluronidase inhibitory activity of solvent extracts of *B. officinalis*. Values are mean ± SD (*n* = 3).

The hyaluronidase inhibitory activities of the six solvent extracts are shown in Figure 6. Unlike the chemical antioxidant capacity assays, the hyaluronidase inhibitory response did not follow exactly the same solvent-dependent pattern as total flavonoid or linarin content. At 2.5 mg/mL, the petroleum ether and 60% ethanol extracts showed relatively strong inhibition, with inhibition rates of approximately 74% and 68%, respectively. At 7.5 mg/mL, the 60% ethanol and 95% ethanol extracts showed high inhibition, reaching approximately 94% and 88%, respectively. These results indicate that both non-polar and medium-polarity fractions of *B. officinalis* may contain constituents relevant to hyaluronidase inhibition. Because no reference inhibitor or IC_50_ determination was included, these data are presented as preliminary comparative screening results rather than benchmarked inhibitory potencies.

### 3.5. Correlation Among Antioxidant Performance Indices

Pearson correlation analysis was used to explore the relationships among the antioxidant performance indices, and the results are summarized in Table 1. ABTS^+^· and DPPH· antioxidant indices were strongly positively correlated (r = 0.985, *p* = 0.002). The correlation between the ABTS^+^· index and ferric reducing power was also positive and reached nominal statistical significance (r = 0.891, *p* = 0.043), whereas the correlation between the DPPH· index and ferric reducing power did not reach significance in this small data set (r = 0.809, *p* = 0.097). Overall, these results indicate that the three antioxidant assays showed generally consistent trends across the solvent fractions, although the degree of consistency differed among assay types. Because the analysis was based on only six solvent fractions, these results should be interpreted as exploratory rather than definitive.

### 3.6. Association of Total Flavonoids and Linarin with Antioxidant Performance

The relationships between the measured compositional variables and antioxidant performance are summarized in Table 2. Both total flavonoid content and linarin content showed positive associations with antioxidant performance across the solvent fractions. Among the observed relationships, linarin showed the strongest association with the ABTS^+^· antioxidant index (r = 0.832). Total flavonoids also showed positive associations with the ABTS^+^·, DPPH·, and ferric reducing power indices, with the strongest observed association being that with ferric reducing power (r = 0.857). These results are directionally consistent with the distribution pattern of the ethanol-rich fractions, which contained higher levels of total flavonoids and linarin and also showed stronger antioxidant performance in the bioassays. Given the limited sample size and the exploratory nature of the analysis, these results are best interpreted as composition–activity trends rather than strong inferential statistical evidence. These findings support the view that phenolic-rich fractions contribute importantly to antioxidant performance, but they do not justify attributing the observed activity to total flavonoids or linarin alone.

### 3.7. Chemical Profiling of the Most Active Extracts

#### 3.7.1. ^1^H NMR Spectroscopic Analysis

The ^1^H NMR spectra were used only as supportive class-level evidence rather than for metabolite-level annotation or semi-quantitative comparison. Clear polarity-dependent spectral differences were observed among the extracts. The aqueous, 60% ethanol, 95% ethanol, and *n*-butanol extracts displayed relatively evident signals in the aromatic and sugar-proton regions, which are consistent with the presence of phenolic glycosides, including flavonoid glycosides and phenylethanoid glycosides. In contrast, the petroleum ether extract was dominated by aliphatic resonances, suggesting enrichment of lipophilic constituents. Because the current NMR analysis did not include two-dimensional NMR confirmation, absolute quantification, or full signal assignment, these spectra are interpreted only as complementary evidence supporting broad polarity-dependent compositional differences. Detailed spectra are provided in the Supplementary Materials.

#### 3.7.2. UPLC–QTOF–MS Analysis and Tentative Compound Identification

**Figure 7:**
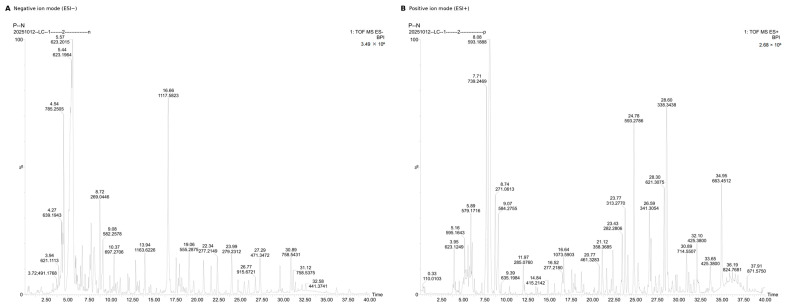
Representative UPLC–QTOF–MS base peak intensity chromatograms of the 60% ethanol extract of *B. officinalis* acquired in negative ion mode ((**A**), ESI−) and positive ion mode ((**B**), ESI+). Putative assignments were based on accurate mass, isotopic pattern, MS/MS fragmentation, and library/literature comparison. Linarin and verbascoside were additionally supported by authentic reference standards. The comprehensive compound list is provided in Table S1.

To provide more direct chemical support for the extract-level activity pattern, the 60% ethanol extract, which showed the strongest overall antioxidant performance in the present study, was further profiled by UPLC–QTOF–MS in both negative and positive ion modes. As shown in Figure 7, the base peak intensity chromatograms revealed a chemically rich profile with multiple well-resolved signals distributed mainly in the early-to-middle retention time range, consistent with the presence of medium-polarity constituents. A set of representative compounds identified or tentatively assigned from this fraction is summarized in Table 3, whereas the comprehensive compound list is provided in the Supplementary Materials (Table S1).

Among the detected constituents, phenylethanoid glycosides and flavonoid glycosides were the dominant classes. Representative phenylethanoid glycosides included echinacoside, acteoside (verbascoside), forsythoside B, and cistanoside A, while representative flavonoid glycosides included linarin, luteolin-7-*O*-glucuronide, baicalin, scutellarin, and isoquercitrin. Chlorogenic acid was also detected as a representative phenolic acid. Among these compounds, linarin and verbascoside were further supported by comparison with authentic reference standards, whereas the remaining assignments were based on accurate mass measurement, isotopic pattern, MS/MS fragmentation behavior, and comparison with library and literature data.

The compositional features of the 60% ethanol extract are consistent with its strong antioxidant performance in the DPPH·, ABTS^+^·, and ferric reducing power assays. In particular, the predominance of medium-polarity phenolic constituents provides direct chemical support for the extract-level composition–activity associations observed in the present study. Although the representative compounds detected in the 60% ethanol extract provide useful chemical context for interpreting the extract-level antioxidant capacity, the current data do not establish the contribution of any single compound to the observed bioactivity. The possible roles of individual constituents remain hypothetical and require activity-guided isolation, purified-compound testing, and potency evaluation in future studies.

## 4. Discussion

The present study establishes a comparative extract-level evidence chain showing that the biological performance of *B. officinalis* is strongly influenced by solvent polarity. Among the six tested extracts, the 60% ethanol fraction showed the strongest overall radical-scavenging activity, whereas the 95% ethanol fraction showed the highest ferric reducing power under the tested conditions. In contrast, hyaluronidase inhibitory activity followed a partially different solvent-dependent pattern, with the petroleum ether extract showing relatively strong inhibition at 2.5 mg/mL and the 60% ethanol and 95% ethanol extracts showing high inhibition at 7.5 mg/mL. Together, these results indicate that different extract classes may contribute differently to antioxidant performance and hyaluronidase inhibition, and that the bioactivity pattern of *B. officinalis* cannot be adequately explained by any single measured compositional parameter alone.

From the perspective of chemical antioxidant capacity, the solvent-dependent trends observed here were broadly consistent with the distribution of total flavonoids and linarin across the six extracts. The ethanol-rich fractions, especially the 60% ethanol and 95% ethanol extracts, contained the highest levels of these measured compositional variables and also showed stronger assay-based antioxidant responses. On a dry-extract basis, these findings support the view that medium- and high-polarity solvents are more effective for obtaining phenolic-rich fractions from *B. officinalis*. This pattern is consistent with the relatively high solubility of flavonoid glycosides and phenylethanoid glycosides in hydroalcoholic solvent systems. Meanwhile, the different rank orders observed among DPPH· scavenging, ABTS^+^· scavenging, and ferric reducing power indicate that chemical antioxidant capacity is assay-dependent and should not be reduced to a single numerical endpoint.

Although the 95% ethanol extract contained the highest total flavonoid and linarin contents, the 60% ethanol extract showed stronger DPPH· and ABTS^+^· radical-scavenging capacity. This discrepancy indicates that the radical-scavenging capacity of the extracts cannot be explained by total flavonoids or linarin alone. The 60% ethanol solvent system may co-extract a broader range of medium-polarity phenolic constituents, including phenylethanoid glycosides, flavonoid glycosides, and phenolic acids, which may contribute additively or synergistically to radical-scavenging. In addition, different antioxidant assays respond differently to electron transfer, hydrogen atom transfer, steric accessibility, and reaction-medium effects. Therefore, the stronger radical-scavenging capacity of the 60% ethanol extract is best interpreted as an extract-level effect resulting from combined chemical features rather than from a single quantified marker.

The comparable hyaluronidase inhibition observed for the petroleum ether and 60% ethanol extracts at 2.5 mg/mL should not be interpreted as evidence for the same active constituents. Rather, it suggests that different chemical classes may contribute to the same enzyme-inhibition endpoint. Hyaluronidase inhibition by plant extracts is commonly used as an in vitro indicator related to anti-inflammatory, anti-allergic, or skin-protective potential, but reference inhibitors and IC_50_ values are usually required to benchmark inhibitory potency [15,16]. In the present study, the 60% ethanol extract was enriched in medium-polarity phenolic constituents, whereas the petroleum ether extract showed predominantly lipophilic features. Lipophilic constituents may interact with enzyme hydrophobic regions, affect substrate–enzyme accessibility, or influence the colorimetric endpoint under the assay conditions. Therefore, the similar inhibition rates of these two extracts may reflect different chemical mechanisms or assay-level effects.

The supportive chemical profiling data provide a compositional context for these extract-level trends. The ^1^H NMR spectra showed broad polarity-dependent spectral differences, with aqueous, 60% ethanol, 95% ethanol, and *n*-butanol extracts displaying relatively evident aromatic and sugar-proton regions, while the petroleum ether extract was dominated by aliphatic resonances. These class-level features are consistent with the different solvent-dependent functional patterns observed in the bioassays. The UPLC–QTOF–MS results further showed that the 60% ethanol extract contained representative phenylethanoid glycosides, flavonoid glycosides, and phenolic acids. These data support the interpretation that the strong radical-scavenging capacity of the 60% ethanol extract is associated with a chemically rich medium-polarity phenolic profile. However, the chemical profiling results provide supportive context rather than direct compound-level activity attribution.

The analytical coverage of the current UPLC–QTOF–MS method should also be considered. ESI-based profiling is more suitable for polar or readily ionizable constituents, whereas non-polar or weakly ionizable compounds may be underrepresented. This is particularly relevant for interpreting the petroleum ether extract, which may contain lipophilic constituents associated with hyaluronidase inhibition but not efficiently detected under the present ESI conditions. Future studies using complementary ionization strategies, such as APCI, together with activity-guided isolation, would be useful for characterizing lipophilic constituents and clarifying their possible contribution to enzyme inhibition.

Another point that should be considered is the basis of comparison. In the present study, extracts were compared on an equal dry-extract mass basis. This approach is useful for evaluating the functional and chemical characteristics of the dried extracts themselves. However, it does not indicate which solvent provides the highest total recovery of active constituents from the original plant material. Because extraction yields may differ substantially among solvents, yield-normalized evaluation would be necessary if the objective were to optimize total activity recovery or raw-material utilization efficiency.

Several points define the scope of interpretation of the present study. First, this work remains a comparative extract-level study and does not provide compound-level potency attribution. Second, the extracts were compared on an equal dry-extract mass basis rather than on an extraction-yield-normalized raw-material basis. Third, ABTS^+^· results were presented as relative scavenging percentages within the current experimental framework rather than as TEAC values. Fourth, hyaluronidase inhibition was expressed only as percentage inhibition because no positive inhibitor control or IC_50_ determination was included. Fifth, total phenolic content was not determined, and the composition–activity analysis was based on selected indicators, namely total flavonoids and linarin. Finally, except for compounds supported by authentic standards, UPLC–QTOF–MS assignments should be regarded as tentative, and ESI-based profiling may underrepresent non-polar or weakly ionizable constituents. These points define the interpretative boundary of the study but do not compromise the value of the solvent-polarity-guided extract-level comparisons.

## 5. Conclusions

This study comparatively evaluated six solvent extracts of *B. officinalis* Maxim. within a unified extract-level framework integrating bioactivity testing, composition–activity association analysis, and supportive chemical profiling. The ethanol-rich fractions, especially the 60% ethanol extract, showed the strongest overall antioxidant performance, while the 95% ethanol extract showed the strongest ferric reducing power under the tested conditions. In the hyaluronidase assay, both the petroleum ether extract and the 60% ethanol extract showed relatively strong inhibitory activity, indicating that non-polar and medium-polarity fractions may both contain relevant constituents.

Supportive ^1^H NMR and UPLC–QTOF–MS data, together with representative chromatographic profiles and representative compound assignments for the 60% ethanol fraction, showed clear polarity-dependent differences in extract composition and helped place the bioactivity results within a more informative chemical context. In particular, the 60% ethanol fraction was characterized by abundant phenylethanoid glycosides and flavonoid glycosides, whereas the petroleum ether fraction showed a predominantly lipophilic profile. Positive composition–activity associations were observed between antioxidant indices and both total flavonoid and linarin contents, although these relationships should be regarded as exploratory rather than definitive evidence of causal contribution.

Overall, the present work provides a solvent-polarity-guided extract-level framework for comparing chemical antioxidant capacity, preliminary hyaluronidase inhibition, and supportive chemical profiles of *B. officinalis* extracts. While compound-level activity attribution requires future positive-control-supported enzyme assays, IC_50_ determination, and activity-guided isolation, the present findings offer a useful basis for fraction selection, extract standardization, and follow-up phytochemical and bioactivity-oriented investigation.

## Figures and Tables

**Table 1:** Correlation among antioxidant performance indices of *B. officinalis* extracts.

Components	DPPH· Index	ABTS^+^· Index	Ferric Reducing Power Index
DPPH· index	1	–	–
ABTS^+^· index	0.985, *p* = 0.002	1	–
Ferric reducing power index	0.809, *p* = 0.097	0.891, *p* = 0.043	1

Note: Data are presented as Pearson correlation coefficients (r) and significance levels (*p*). The analysis was performed across the six solvent fractions (*n* = 6). Because of the small sample size, these results should be interpreted as exploratory rather than definitive. The dash (–) indicates that a value is not applicable because only the lower triangular part of the symmetric correlation matrix is shown.

**Table 2:** Association of total flavonoids and linarin with antioxidant performance indices of *B. officinalis* extracts.

Component	ABTS^+^· Index	DPPH· Index	Ferric Reducing Power Index
Total flavonoids	0.736	0.682	0.857
Linarin	0.832	0.812	0.830

Note: Values are Pearson correlation coefficients (r). Because the analysis was based on a small number of extract fractions, these results are presented as exploratory associations rather than strong inferential statistical evidence.

**Table 3:** Representative compounds identified or tentatively assigned in the 60% ethanol extract of *B. officinalis* by UPLC–QTOF–MS.

No.	Compound	Molecular Formula	Rt (min)	Observed *m*/*z* (Adduct)	Ion Mode	Compound Class	Identification Level	Possible Relevance
1	Echinacoside	C_35_H_46_O_20_	4.54	785.2525 [M − H]−	ESI−	Phenylethanoid glycoside	Tentative	Phenolic constituent potentially relevant to antioxidant activity
2	Acteoside (Verbascoside)	C_29_H_36_O_15_	5.95	623.1973 [M − H]−	ESI−	Phenylethanoid glycoside	Supported by reference standard	Phenylethanoid glycoside associated with antioxidant potential
3	Forsythoside B	C_34_H_44_O_19_	5.26	755.2401 [M − H]−	ESI−	Phenylethanoid glycoside	Tentative	Medium-polarity phenolic glycoside relevant to extract-level antioxidant effects
4	Cistanoside A	C_36_H_48_O_20_	5.19	799.2636 [M − H]−	ESI−	Phenylethanoid glycoside	Tentative	Phenylethanoid glycoside contributing to the phenolic-rich profile
5	Linarin	C_28_H_32_O_14_	8.08	593.1865 [M + H]+	ESI+	Flavonoid glycoside	Supported by reference standard	Major quantified marker associated with antioxidant performance
6	Luteolin-7-*O*-glucuronide	C_21_H_18_O_12_	4.84	461.0720 [M − H]−	ESI−	Flavonoid glycoside	Tentative	Flavonoid glycoside consistent with radical-scavenging activity
7	Baicalin	C_21_H_18_O_11_	5.81	445.0778 [M − H]−	ESI−	Flavonoid glycoside	Tentative	Flavonoid glycoside contributing to the antioxidant-active fraction
8	Scutellarin	C_21_H_18_O_12_	3.96	621.1107 [M − H]−	ESI−	Flavonoid glycoside	Tentative	Representative polar flavonoid glycoside
9	Isoquercitrin	C_21_H_20_O_12_	4.39	463.0876 [M − H]−	ESI−	Flavonoid glycoside	Tentative	Phenolic glycoside with potential antioxidant relevance
10	Chlorogenic acid	C_16_H_18_O_9_	2.30	353.0871 [M − H]−	ESI−	Phenolic acid	Tentative	Representative phenolic acid supporting the phenolic-rich composition

Note: Rt, retention time. Compound assignments were based on accurate mass measurement, isotopic pattern, MS/MS fragmentation, and comparison with library/literature data. Linarin and verbascoside (acteoside) were further supported by comparison with authentic reference standards, whereas the remaining assignments are tentative. The table presents representative compounds selected from the comprehensive compound list provided in the Supplementary Materials (Table S1).

## Data Availability

The data presented in this study are available in the article and Supplementary Materials. Additional supporting information may be provided by the corresponding author upon reasonable request.

## Supplementary Materials

The following supporting information can be downloaded at: https://www.mdpi.com/article/10.3390/molecules31101706/s1, The Supporting Information includes ^1^H NMR spectra of representative extracts (Figures S1–S6), UPLC–QTOF–MS base peak intensity chromatograms of the 60% ethanol extract acquired in negative and positive ion modes (Figures S7 and S8), a comprehensive compound list for the 60% ethanol extract (Table S1), and a brief statement on the limitations of supporting chemical profiling.
