# Supplementary material for: Solvent Polarity Shapes Antioxidant Capacity, Preliminary Hyaluronidase Inhibition, and Chemical Profile of Buddleja officinalis Extracts
Source: Molecules. 2026 May 18;31(10):1706. doi: 10.3390/molecules31101706 (PMC13209219; doi:10.3390/molecules31101706)
Supplement: Supplementary file 1 [file molecules-31-01706-s001.zip › molecules-4281969-supplementary.pdf]

Supporting Information

for

Solvent Polarity Shapes Antioxidant Capacity, Preliminary Hyaluronidase Inhibition, and Chemical Profiles of *Buddleja officinalis* Extracts

Gang Tian[1,2,3,*], Yihang Tian[1,3], Shiping Cheng[1,2], Cong Yang[4], Yongjun Han[1,3]

[1] School of Chemistry and Chemical Engineering, Pingdingshan University, Pingdingshan, Henan 467000, China

[2] Henan Key Laboratory of Germplasm Innovation and Utilization of Eco-Economic Woody Plant, Pingdingshan, Henan 467000, China

[3] Yaoshan Laboratory, Pingdingshan University, Pingdingshan, Henan 467000, China

[4] School of Mathematics and Statistics, Pingdingshan University, Pingdingshan, Henan 467000, China

* Correspondence: gangty@126.com

S1. ^{1}H NMR Spectroscopic Analysis

^{1}H NMR spectra were recorded for representative extracts of different polarity, including the aqueous, 60% ethanol, 95% ethanol, ethyl acetate, *n*-butanol, and petroleum ether extracts of *Buddleja officinalis*. The NMR data were used only as supportive class-level compositional evidence for comparing broad chemical features among extracts prepared with different solvents. These spectra were not used for complete metabolite-level annotation, semi-quantitative comparison, or direct activity attribution.

S1.1. Experimental Parameters

Pulse sequence: zg30

Number of scans: 8

Relaxation delay: 1.0 s

Acquisition time: 2.04 s

Solvent: DMSO-d_6

Temperature: 297.3 K

Spectrometer: Bruker AVANCE 400 MHz NMR spectrometer

S1.2. Figures

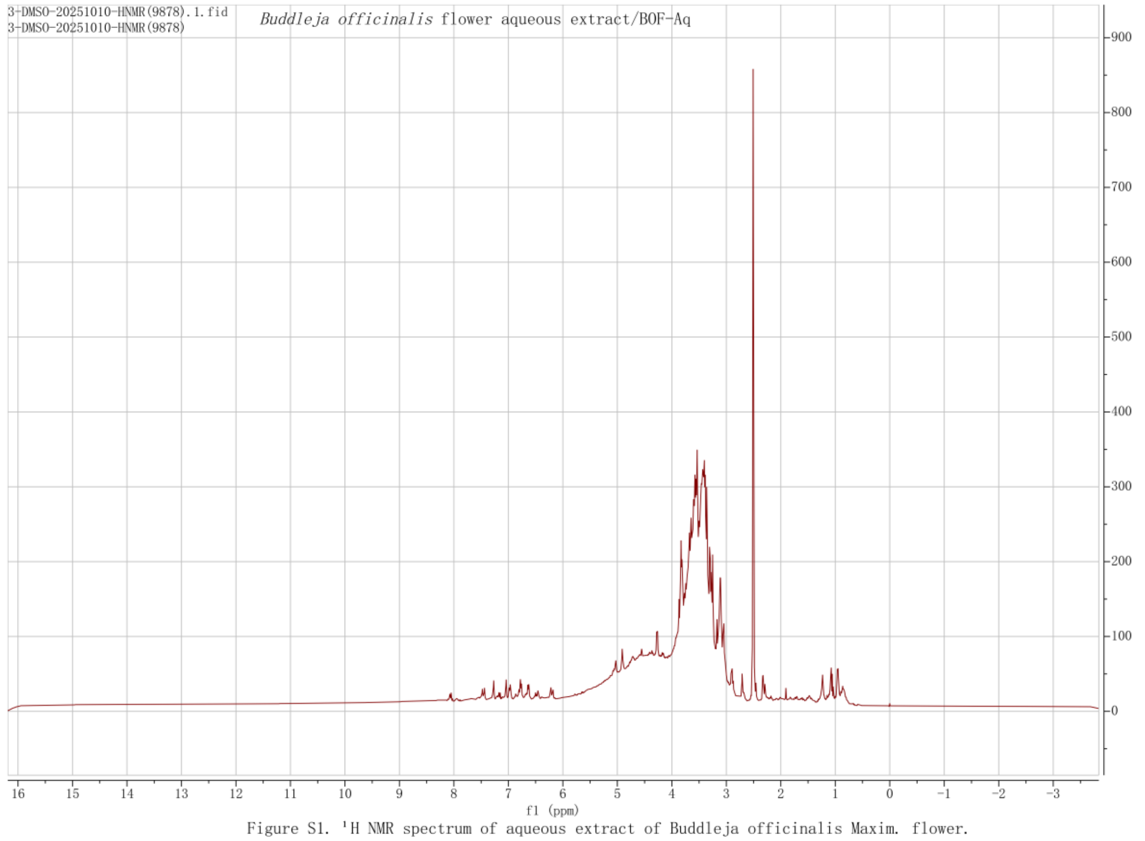

Figure S1. ^{1}H NMR spectrum of the aqueous extract of *B. officinalis*.

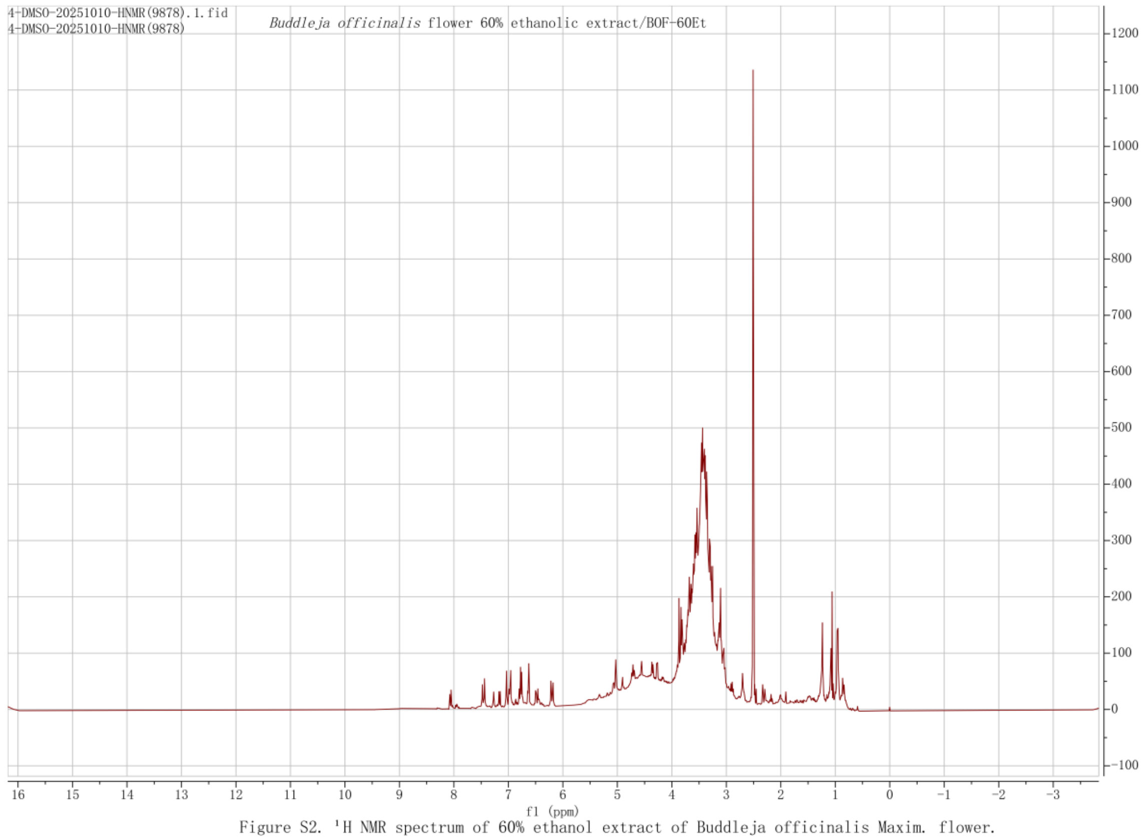

Figure S2. ^{1}H NMR spectrum of the 60% ethanol extract of *B. officinalis*.

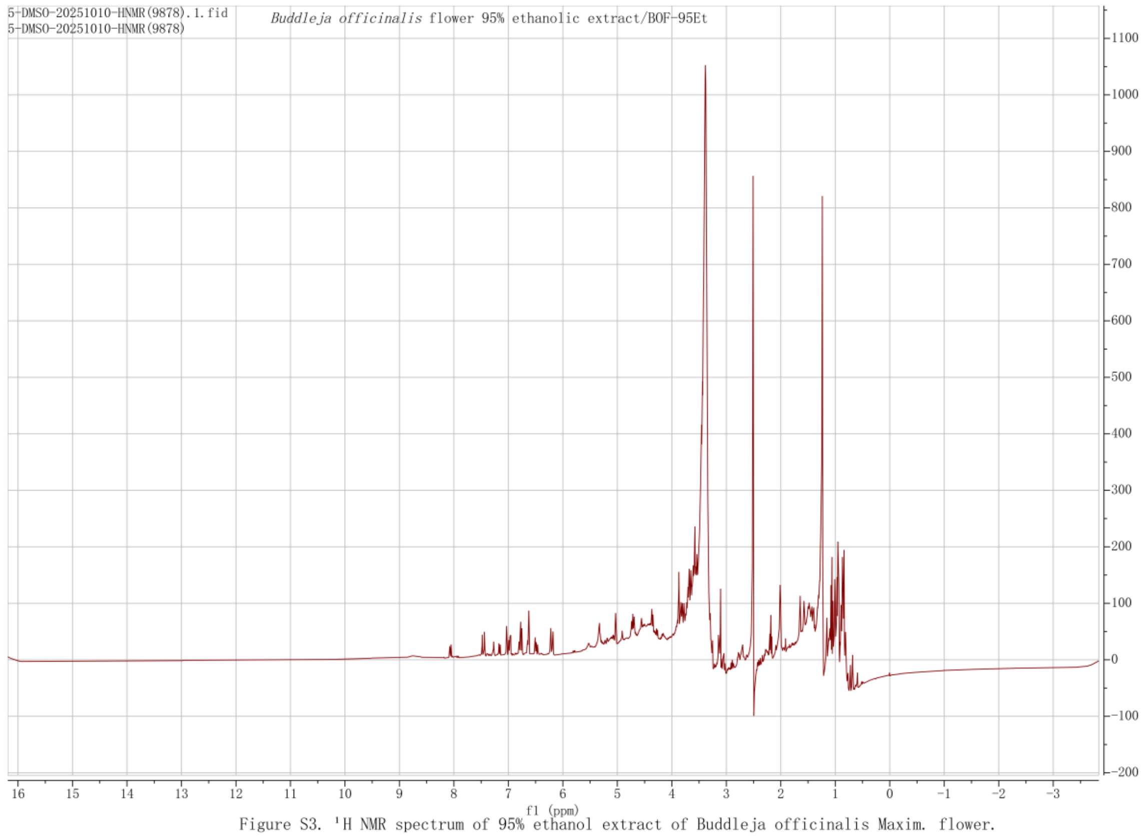

Figure S3. ^{1}H NMR spectrum of the 95% ethanol extract of *B. officinalis*.

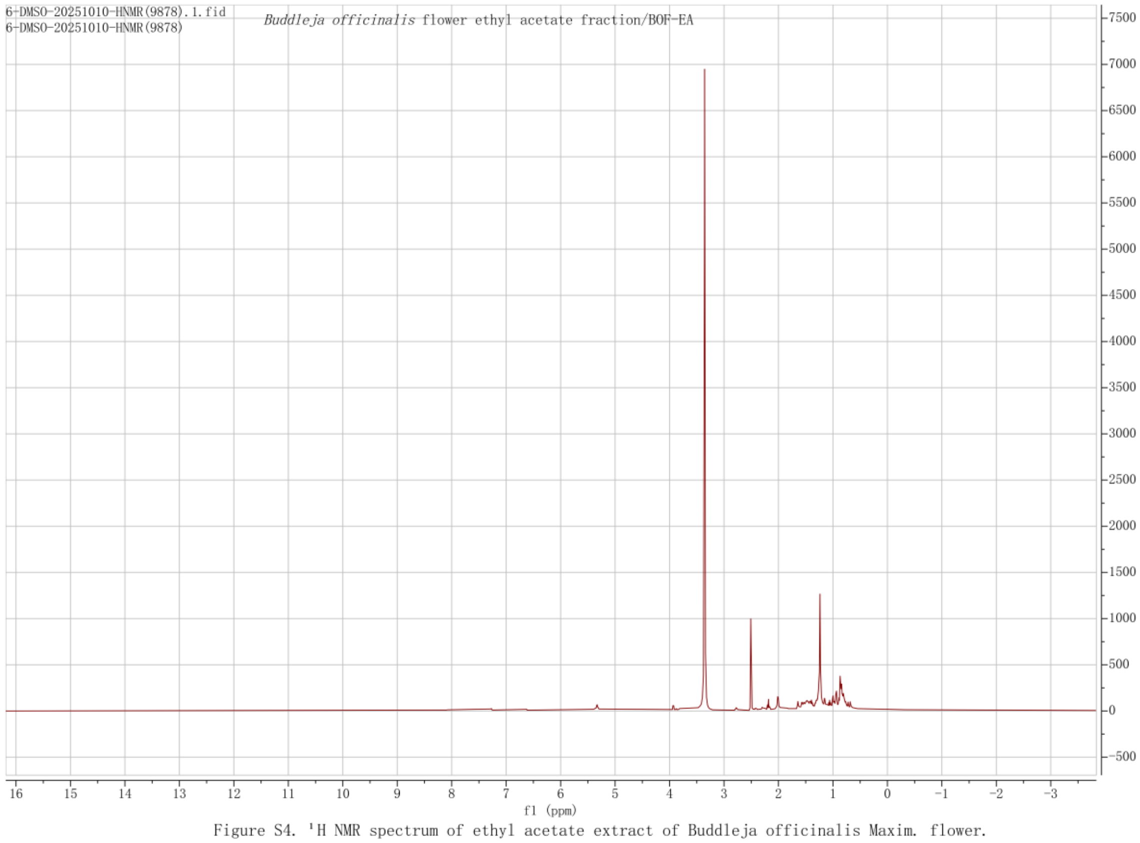

Figure S4. ^{1}H NMR spectrum of the ethyl acetate extract of *B. officinalis*.

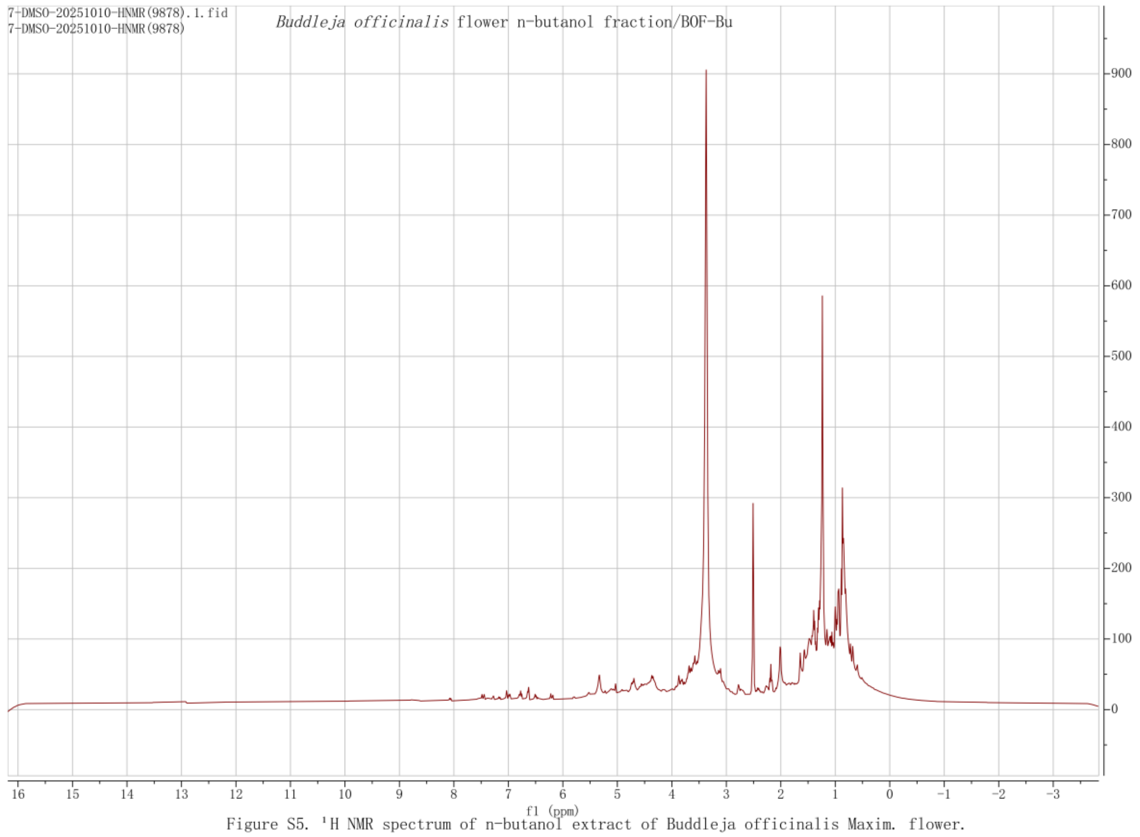

Figure S5. ^{1}H NMR spectrum of the *n*-butanol extract of *B. officinalis*.

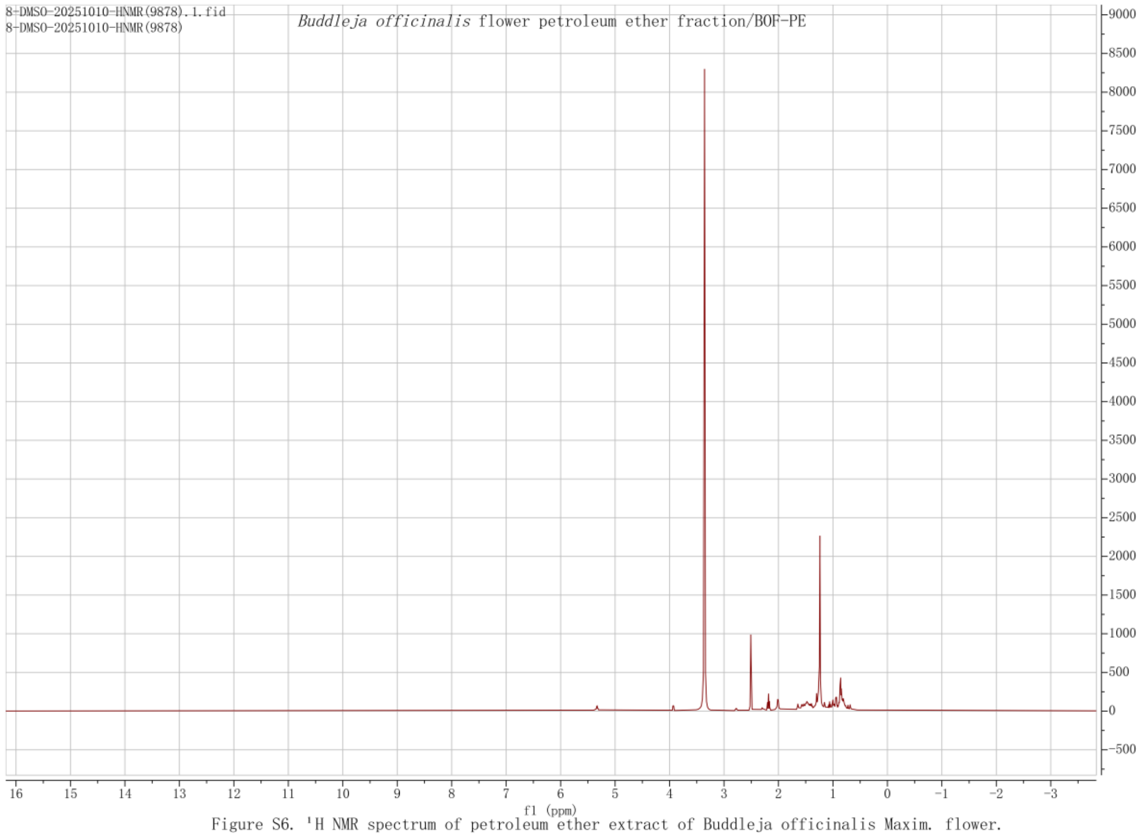

Figure S6. ^{1}H NMR spectrum of the petroleum ether extract of *B. officinalis*.

Note: The ^{1}H NMR spectra are provided as supplementary visual evidence for broad polarity-dependent compositional differences among extracts and were not used for complete metabolite-level annotation, semi-quantitative comparison, or direct activity attribution.

S2. UPLC–QTOF–MS Conditions and Chromatograms

Chemical profiling was performed using the 60% ethanol extract of *B. officinalis*, which showed the strongest overall radical-scavenging capacity in the main study. Representative base peak intensity chromatograms acquired in negative and positive ion modes are shown in Figures S7 and S8, respectively. These chromatograms provide supplementary visual support for the representative compound assignments summarized in Table S1.

S2.1. Chromatographic and Mass Spectrometric Conditions

Chemical profiling was performed using a Waters ACQUITY H-Class UPLC system coupled with a Waters Xevo G2-XS QTOF mass spectrometer equipped with an electrospray ionization source.

Chromatographic conditions

Separation was carried out on a Waters BEH C18 column (2.1 × 100 mm, 1.7 μm) maintained at 40 °C. The mobile phase consisted of 0.1% formic acid in water (A) and 0.1% formic acid in acetonitrile (B), delivered at a flow rate of 0.4 mL/min. The gradient elution program was as follows: 0–1 min, 5% B; 1–35 min, 5–98% B; 35–37 min, 98% B; and 37.1–40 min, 5% B for re-equilibration. The injection volume was 2 μL.

Mass spectrometric conditions

Data were acquired in both positive and negative ESI modes. The capillary voltage was set at 2.0 kV in ESI+ mode and 1.8 kV in ESI− mode. The source temperature was 110 °C, the desolvation temperature was 400 °C, and the desolvation gas flow was 800 L/h. The mass range was m/z 50–1200. MS/MS data were obtained using a collision energy ramp of 20–40 eV.

S2.2. Representative Base Peak Intensity Chromatograms

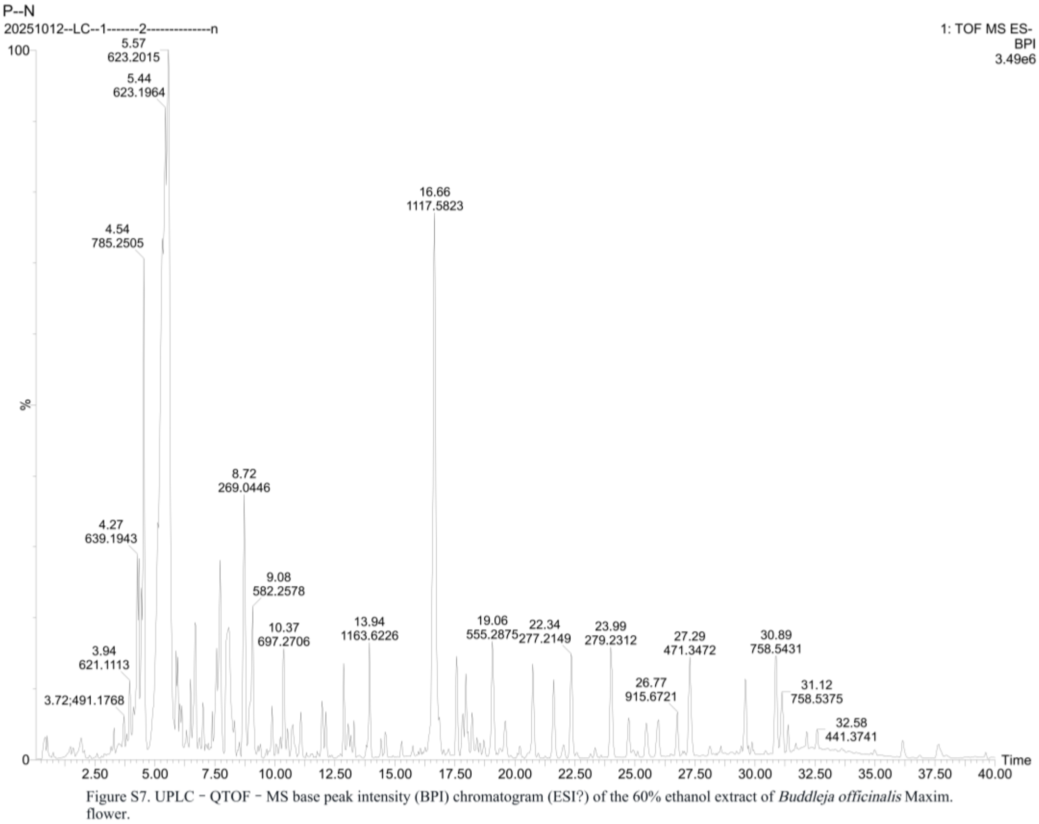

Figure S7. UPLC–QTOF–MS base peak intensity chromatogram of the 60% ethanol extract of *B. officinalis* acquired in negative ion mode (ESI−).

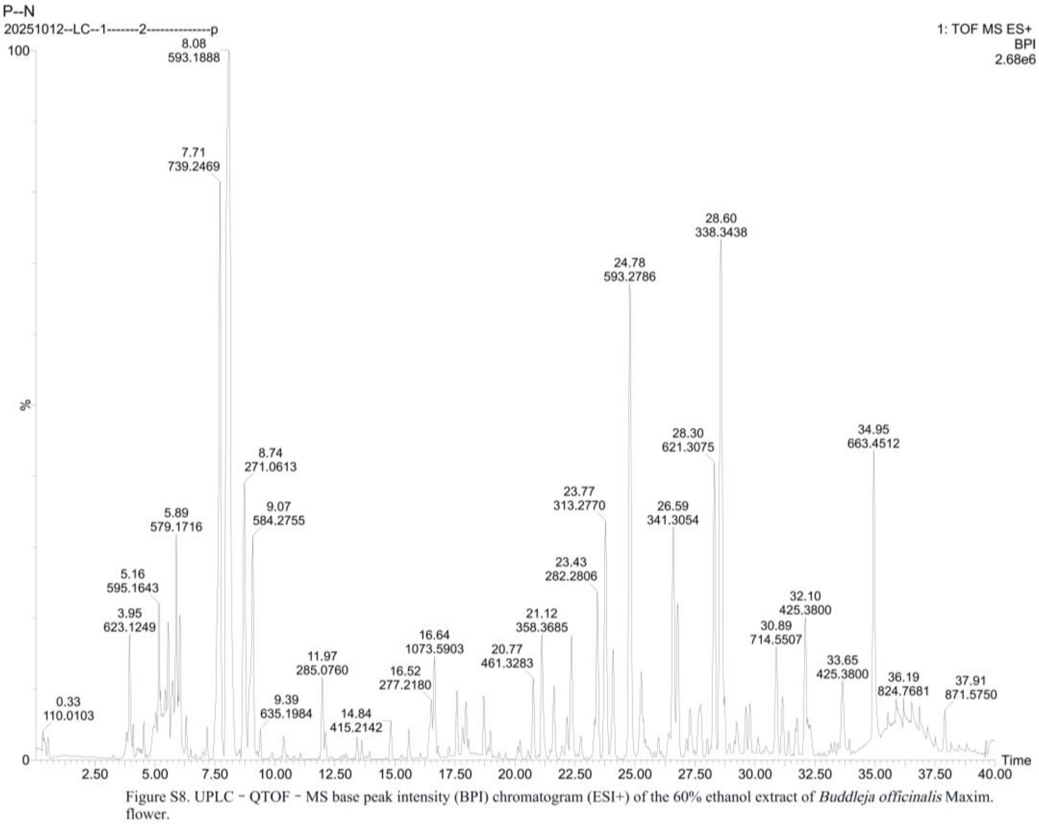

Figure S8. UPLC–QTOF–MS base peak intensity chromatogram of the 60% ethanol extract of *B. officinalis* acquired in positive ion mode (ESI+).

S3. Tentative Compound Identification by UPLC–QTOF–MS

S3.1. Identification Methodology

Compounds were tentatively identified by matching accurate mass data, typically within 5 ppm, isotopic patterns, and MS/MS fragmentation features with entries in the Waters Traditional Medicine Library 2.0 and relevant published data on *B. officinalis* and related medicinal plants. For the key marker compounds quantified or discussed in the main text, namely linarin and verbascoside (acteoside), identification was further supported by comparison of retention time and MS/MS spectra with those of authentic commercial reference standards.

All other compound assignments should be regarded as tentative. The UPLC–QTOF–MS data were used to provide supportive chemical context for extract-level interpretation rather than to establish definitive compound-level bioactivity attribution.

S3.2. Comprehensive Compound List

Table S1. Comprehensive list of representative compounds tentatively identified in the 60% ethanol extract of *B. officinalis* by UPLC–QTOF–MS.

No.	Tentative Identification	Molecular Formula	Rt (min)	Observed m/z (Adduct)	Error (ppm)	Response	Ion Mode	Compound Class
1	Echinacoside	$C_{35}H_{46}O_{20}$	4.54	785.2525 [M−H]−	2.0	780639	ESI−	Phenylethanoid glycoside
2	Echinacoside	$C_{35}H_{46}O_{20}$	4.54	804.2896 M+NH4]+	-3.1	11731	ESI+	Phenylethanoid glycoside
3	Acteoside (Verbascoside)	$C_{29}H_{36}O_{15}$	5.95	623.1973 [M−H]−	-1.3	172696	ESI−	Phenylethanoid glycoside
4	Acteoside	$C_{29}H_{36}O_{15}$	0.41	663.1677 [M+K]+	-1.3	16361	ESI+	Phenylethanoid glycoside
5	Forsythoside B	$C_{34}H_{44}O_{19}$	5.26	755.2401 [M−H]−	-0.4	157690	ESI−	Phenylethanoid glycoside
6	Cistanoside A	$C_{36}H_{48}O_{20}$	5.19	799.2636 [M−H]−	-3.8	29614	ESI−	Phenylethanoid glycoside
7	Linarin	$C_{28}H_{32}O_{14}$	8.08	593.1865 [M+H]+	0.0	2646308	ESI+	Flavonoid glycoside
8	Linarin	$C_{28}H_{32}O_{14}$	5.18	593.1517 [M+HCOO]−	0.8	408476	ESI−	Flavonoid glycoside
9	Luteolin-7-O-glucuronide	$C_{21}H_{18}O_{12}$	4.84	461.0720 [M−H]−	-1.1	45275	ESI−	Flavonoid glycoside
10	Luteolin-7-O-glucuronide	$C_{21}H_{18}O_{12}$	4.84	463.0868 [M+H]+	-0.7	25424	ESI+	Flavonoid glycoside
11	Baicalin	$C_{21}H_{18}O_{11}$	5.81	445.0778 [M−H]−	0.4	58960	ESI−	Flavonoid glycoside
12	Baicalin	$C_{21}H_{18}O_{11}$	5.81	447.0924 [M+H]+	0.4	107946	ESI+	Flavonoid glycoside
13	Scutellarin	$C_{21}H_{18}O_{12}$	3.96	621.1107 [M−H]−	1.6	139181	ESI−	Flavonoid glycoside
14	Scutellarin	$C_{21}H_{18}O_{12}$	3.96	623.1241 [M+H]+	-0.3	167636	ESI+	Flavonoid glycoside

No.	Tentative Identification	Molecular Formula	Rt (min)	Observed m/z (Adduct)	Error (ppm)	Response	Ion Mode	Compound Class
15	Isoquercitrin	$C_{21}H_{20}O_{12}$	4.39	463.0876 [M−H]−	-1.3	44310	ESI−	Flavonoid glycoside
16	Daidzein-4′,7-di-O-glucoside	$C_{27}H_{30}O_{14}$	5.71	577.1570 [M−H]−	1.2	110242	ESI−	Isoflavone glycoside
17	Daidzein-4′,7-di-O-glucoside	$C_{27}H_{30}O_{14}$	5.71	579.1707 [M+H]+	-0.3	198702	ESI+	Isoflavone glycoside
18	Cyanidin-3-O-glucoside	$C_{21}H_{20}O_{11}$	5.02	447.0933 [M−H]−	0.0	93695	ESI−	Anthocyanin
19	Cyanidin-3-O-glucoside	$C_{21}H_{20}O_{11}$	5.05	449.1079 [M+H]+	0.1	84621	ESI+	Anthocyanin
20	Quercetin-7-O-rutinoside	$C_{27}H_{30}O_{16}$	4.41	609.1455 [M−H]−	-0.9	13379	ESI−	Flavonoid glycoside
21	Apigenin-7-O-glucuronide	$C_{22}H_{20}O_{10}$	6.13	489.1037 [M+HCOO]−	-0.2	28767	ESI−	Flavonoid glycoside
22	Kaempferol-3-O-glucuronide methyl ester	$C_{22}H_{20}O_{12}$	6.13	475.0874 [M−H]−	-1.8	7591	ESI−	Flavonoid glycoside
23	Chlorogenic acid	$C_{16}H_{18}O_{9}$	2.30	353.0871 [M−H]−	-1.9	10329	ESI−	Phenolic acid
24	Ferulic acid glucoside	$C_{16}H_{20}O_{9}$	3.29	355.1029 [M−H]−	-1.7	8662	ESI−	Phenolic acid glycoside
25	1-Hydroxyanthraquinone	$C_{14}H_{8}O_{3}$	8.73	269.0459 [M+HCOO]−	1.3	260700	ESI−	Quinone
26	Betulinic acid	$C_{30}H_{48}O_{3}$	22.28	455.3518 [M−H]−	-2.7	14791	ESI−	Triterpenoid
27	Betulinic acid	$C_{30}H_{48}O_{3}$	22.28	457.3666 [M+H]+	-2.3	8008	ESI+	Triterpenoid
28	2α-Hydroxyursolic acid	$C_{30}H_{48}O_{4}$	28.49	473.3620 [M+H]+	-1.1	45394	ESI+	Triterpenoid
29	Diosgenin	$C_{27}H_{42}O_{3}$	20.76	459.3113 [M+HCOO]−	-0.7	132852	ESI−	Steroidal sapogenin
30	Diosgenin	$C_{27}H_{42}O_{3}$	20.76	415.3204 [M+H]+	-0.6	14857	ESI+	Steroidal sapogenin
31	Stigmast-4-ene-3,6-dione	$C_{29}H_{46}O_{2}$	25.98	471.3477 [M+HCOO]−	-0.5	72053	ESI−	Steroid
32	Stigmast-4-ene-3,6-dione	$C_{29}H_{46}O_{2}$	25.99	427.3574 [M+H]+	0.7	32679	ESI+	Steroid
33	Campesterol glucoside	$C_{34}H_{58}O_{6}$	16.65	585.4133 [M+Na]+	1.2	7090	ESI+	Steroid glycoside
34	Linolenic acid	$C_{18}H_{30}O_{2}$	17.97	279.2329 [M+H]+	3.9	73635	ESI+	Fatty acid
35	Linolenic acid	$C_{18}H_{30}O_{2}$	22.64	277.2165 [M−H]−	-2.8	12037	ESI−	Fatty acid
36	Palmitic acid	$C_{16}H_{32}O_{2}$	32.98	255.2320 [M−H]−	-3.7	5324	ESI−	Fatty acid
37	Butyl stearate	$C_{22}H_{44}O_{2}$	21.13	358.3683 [M+NH4]+	1.1	161921	ESI+	Fatty acid ester
38	Monopalmitin	$C_{19}H_{38}O_{4}$	23.78	353.2666 [M+Na]+	1.1	54333	ESI+	Monoglyceride
39	Maltose	$C_{12}H_{22}O_{11}$	0.41	381.0797 [M+K]+	0.7	30507	ESI+	Carbohydrate
40	Adenosine	$C_{10}H_{13}N_{5}O_{4}$	0.52	268.1046 [M+H]+	2.1	6480	ESI+	Nucleoside
41	Citric acid	$C_{6}H_{8}O_{7}$	0.51	191.0198 [M−H]−	0.4	20164	ESI−	Organic acid
42	Catenarin	$C_{15}H_{10}O_{6}$	7.58	287.0561 [M+H]+	3.9	123692	ESI+	Anthraquinone
43	1,3,6-Trihydroxy-2-methylanthraquinone	$C_{15}H_{10}O_{5}$	8.73	271.0615 [M+H]+	5.1	371111	ESI+	Anthraquinone

No.	Tentative Identification	Molecular Formula	Rt (min)	Observed m/z (Adduct)	Error (ppm)	Response	Ion Mode	Compound Class
44	Escin IVa	$C_{55}H_{86}O_{24}$	9.08	1165.5258 [M+Cl]−	4.7	59985	ESI−	Triterpenoid saponin
45	Saikosaponin E	$C_{42}H_{68}O_{12}$	16.64	765.4761 [M+H]+	-2.9	29544	ESI+	Triterpenoid saponin
46	Oleanolic acid derivative	$C_{35}H_{58}O_{9}$	33.62	622.4081 [M+H]+	–	6856	ESI+	Triterpenoid
47	Betulin	$C_{30}H_{50}O_{2}$	32.60	441.3736 [M−H]−	-0.4	61108	ESI−	Triterpenoid
48	(24Z)-27-Hydroxy-7,24-tirucalladien-3-one	$C_{30}H_{48}O_{2}$	25.19	441.3722 [M+H]+	-1.2	35125	ESI+	Triterpenoid
49	3,11-Dioxo-β-amyrin	$C_{30}H_{46}O_{2}$	27.71	439.3574 [M+H]+	0.7	155377	ESI+	Triterpenoid
50	Taraxerone	$C_{30}H_{48}O$	33.25	425.3775 [M+H]+	-0.7	15610	ESI+	Triterpenoid

Notes: Rt, retention time. Assignments were based on accurate mass, isotopic pattern, MS/MS fragmentation, the Waters Traditional Medicine Library 2.0, and relevant literature. Only linarin and verbascoside (acteoside) were confirmed with authentic standards; all other assignments are tentative. Duplicate ion-mode entries are listed separately. These data are provided as supplementary chemical profiling information and do not establish direct compound-level bioactivity contribution.
